# *GmFULa* improves soybean yield by enhancing carbon assimilation without altering flowering time or maturity

**DOI:** 10.1007/s00299-021-02752-y

**Published:** 2021-07-16

**Authors:** Yanlei Yue, Shi Sun, Jiawen Li, Haidong Yu, Hongxia Wu, Baiquan Sun, Tao Li, Tianfu Han, Bingjun Jiang

**Affiliations:** 1 College of Life Sciences, Henan Agricultural University, Zhengzhou, 450002 China; 2 MARA Key Lab of Soybean Biology (Beijing), Institute of Crop Sciences, The Chinese Academy of Agricultural Sciences, Beijing, 100081 China

**Keywords:** Soybean (*Glycine max* (L.) Merr.), *GmFULa*, Yield, Biomass, Palisade tissue, Sucrose synthesis, Transport

## Abstract

**Key message:**

***GmFULa improved soybean yield by enhancing carbon assimilation. Meanwhile, different from known yield-related genes, it did not alter flowering time or maturity.***

**Abstract:**

Soybean (*Glycine max* (L.) Merr.) is highly demanded by a continuously growing human population. However, increasing soybean yield is a major challenge. *FRUITFULL* (*FUL*), a MADS-box transcription factor, plays important roles in multiple developmental processes, especially fruit and pod development, which are crucial for soybean yield formation. However, the functions of its homologs in soybean are not clear. Here, through haplotype analysis, we found that one haplotype of the soybean homolog *GmFULa* (*GmFULa-H02*) is dominant in cultivated soybeans, suggesting that *GmFULa-H02* was highly selected during domestication and varietal improvement of soybean. Interestingly, transgenic overexpression of *GmFULa* enhanced vegetative growth with more biomass accumulated and ultimately increased the yield but without affecting the plant height or changing the flowering time and maturity, indicating that it enhances the efficiency of dry matter accumulation. It also promoted the yield factors like branch number, pod number and 100-seed weight, which ultimately increased the yield. It increased the palisade tissue cell number and the chlorophyll content to promote photosynthesis and increase the soluble sugar content in leaves and fresh seeds. Furthermore, GmFULa were found to be sublocalized in the nucleus and positively regulate sucrose synthases (*SUSs*) and sucrose transporters (*SUTs*) by binding with the conserved CArG boxes in their promoters. Overall, these results showed *GmFULa* promotes the capacity of assimilation and the transport of the resultant assimilates to increase yield, and provided insights into the link between *GmFULa* and sucrose synthesis with transport-related molecular pathways that control seed yield.

**Supplementary Information:**

The online version contains supplementary material available at 10.1007/s00299-021-02752-y.

## Introduction

Soybean (*Glycine max* (L.) Merr.) provides large amounts of edible oils and vegetable proteins for humans and livestock, and thus, demand for soybean is increasing globally due to human population growth. However, available cultivated land resources are largely limited, meaning that increasing soybean unit yield, as a way to increase the total yield, is a major challenge. Soybean yield is based on pod number per plant, seed number per pod and 100-seed weight (Van Roekel et al. 2015; Yan et al. 2017; Bianchi et al. 2020). Taking into account that soybean is sensitive to photoperiod, maturity loci have important roles in yield. However, there are many yet unknown quantitative loci reported to be linked to yield-related traits in SoyBase (https://www.soybase.org/) but only a few loci have been molecularly identified. Among them, *GmCYP78A10* is related to pod number and 100-seed weight and *Ln* is a major gene controlling four-seed pod development in soybean (Jeong et al. 2012; Wang et al. 2015; Sayama et al. 2017). *GsCID1* is responsible for 100-seed weight in wild soybean (Hu et al. 2020). Recently, *GmKIX8-1* was demonstrated to be associated with soybean seed weight (Nguyen et al. 2021). However, the molecular mechanism controlling yield in soybean is largely unknown.

*FRUITFULL* (*FUL*), a MADS-box transcription factor, has essential and pleiotropic roles in multiple developmental processes including shoot initiation, reproductive transition, inflorescence differentiation and fruit development (Ferrandiz et al. 2000; Balanza et al. 2019; Maheepala et al. 2019; Zhang et al. 2019a; Zhao et al. 2019). In Arabidopsis, *FUL* (*AGL8*) is the main member of the regulatory network that determines fruit growth pattern (Mandel and Yanofsky 1995; Alvarez-Buylla et al. 2019). *FUL* down-regulates *APETALA2* and *INDEHISCENT*, and they two promote pod elongation (Mandel and Yanofsky 1995; Balanza et al. 2019; Di Marzo et al. 2020). *FUL* is necessary for terminal flower formation in seedless states (Balanza et al. 2019). *FUL* also contributes to the differentiation of inflorescence, stem, leaf and carpel (Zhang et al. 2013; Yao et al. 2019). Similar functions were found in the pea and cotton homologous genes *VEGETATIVE1* and *GhMADS22*, respectively (Berbel et al. 2012; Zhang et al. 2013). *CsFUL1* regulates fruit length in cucumber, and *DEFH28*, a homologous gene in *Antirrhinum japonicum*, regulates carpel wall differentiation and fruit ripening (Müller et al. 2001; Zhao et al. 2019). In tomato, *TDR4*/*FUL1* and *MBP7*/*FUL2* are involved in cell wall modification and affect fruit ripening without dependence on ethylene (Bemer et al. 2012; Li et al. 2019). FUL can also modulate plant architecture. Rice homolog *OsMADS18* is negatively related to the number of tillers, and controls the branch angle by inhibiting *SAUR10* (*SMALL AUXIN UPREGULATED RNA 10*) expression (Bemer et al. 2017).

In addition, FUL functions in the formation of secondary metabolites. *VmTDR4* is involved in the accumulation of anthocyanins during the normal ripening period in blueberries, and *DR4*/*FUL1* and *MBP7*/*FUL2* participate in the synthesis of volatile substances in tomato fruits (Jaakola et al. 2010; Bemer et al. 2012) and affect the accumulation of pigment in tomato maturity (Wang et al. 2014). Moreover, in Arabidopsis, FUL can intertalk with hormone- and light-signaling pathways. It directly regulates cytokinin oxidase genes *CKX5* and *CKX6*, plant pigment interaction factor *PIL1*, della family genes *RGL2* and *GAI*, and auxin-responsive gene *SAUR10* (Bemer et al. 2017; Di Marzo et al. 2020). FUL protein can bind to the CArG box in the promoter of the *SHP* gene to regulate the development of fruit petal and embryo and limit pod dehiscence of *Arabidopsis thaliana* by regulating cell proliferation (Sehra and Franks 2017). In soybean, Jia et al. (2015) found eight *AP1*/*FUL*-like genes: *GmFULa* (*Glyma.06G205800*); *GmFULb* (*Glyma.04G159300*); *GmFULc* (*Glyma.05G018800*); *GmFULd* (*Glyma.17G081200*); *GmAP1a* (*Glyma.16G091300*); *GmAP1b* (*Glyma.08G269800*); *GmAP1c* (*Glyma.01G064200*); and *GmAP1d* (*Glyma.02G121600*). Few studies have been performed on these soybean *FUL* homologs, except that *GmFULa* was found to be highly expressed in the root and shoot apices and might be involved in plant architecture and yield. Particularly, the function of *GmFULa* and its mode of action are unclear.

Here, we analyzed the molecular function of *GmFULa.* First, we found that it has six haplotypes with *GmFULa-H01* and *GmFULa-H02* dominant. *GmFULa-H01* is dominant in wild soybeans, while *GmFULa-H02* is dominant in cultivated soybeans especially southern cultivars, indicating that it is an elite allele for soybean breeding. Overexpression of *GmFULa-H02* results in more biomass and higher yield with increased seed number and weight. We further show that whole-soluble sugar contents were increased in fresh leaves and seeds. Consistently, GmFULa bound to the promoters of *GmSUS12* and *GmSUT5* and activated their expression. The cell distribution in palisade tissue regulated by *GmFULa* and chlorophyll content increased significantly with high photosynthetic efficiency in an overexpression line. Our results show that the *GmFULa*–*GmSUSs*/*GmSUTs* pathway regulates the seed yield of soybean, controls the cell number of palisade tissue, and enhances organic matter accumulation by increasing whole-soluble sugar contents in leaf tissue. In contrast, it does not affect flowering time or maturity.

## Materials and methods

### Cloning and sequence analysis of *GmFULa*

The sequences of *GmFUL* family genes and their encoding proteins were obtained from Phytozome (https://phytozome.jgi.doe.gov). Conserved domains were searched in the NCBI database. The CDS of *GmFULa* was amplified from the soybean cultivar Zigongdongdou (ZGDD) with the primers listed in Supplementary Table S1. Then, the PCR products were cloned into the pZeroBack/blunt vector (TianGen, Beijing, China). Sanger sequencing was then performed to confirm the sequences and variations (Shanghai Sangon Biological Engineering Technology and Service CO., LTD, Zhengzhou, China).

### Haplotype analysis of *GmFULa*

Publicly available genome resequencing data (NCBI: SRP062560, SRP045129) were used. These sequences were mapped to the Williams 82 genome (v275) using bwa v0.7.10 with default parameters. SNPs/indels were called using the UnifiedGenotyper module (-stand_call_conf 30.0 -stand_emit_conf 10.0) of the GenomeAnalysisTK suite (https://gatk.broadinstitute.org/) (Zhang et al. 2019b). The polymorphism information of *GmFULa* was further extracted. The SNP/InDel sites located in the CDS region and including missense mutations were selected to define haplotypes and perform haplotype analysis (Jiang et al. 2019).

### Plant materials and growth conditions

ZGDD is a photoperiod-sensitive cultivar suitable of low latitude conditions. In this study, ZGDD was used as a wild-type control for molecular analysis including genetic transformation. To investigate soluble sugar content, chlorophyll content, sucrose synthase activity, biomass, cell morphology and gene expression, plants were grown in pots containing a 1:1 mixture of forest vermiculite in a light chamber (20000 Lux) under short-day condition (12 h/12 h) at 25 °C with 60% humidity.

Plants for the investigation of maturity, yield-related traits and photosynthetic activity were grown under field conditions (with 1.5 m row length, 75 cm row spacing, 10 cm plant spacing, three replicates and completely randomized design) from November of 2016, 2019 and 2020 to next Aprils in tropical city of Sanya (18.1° N, 109.2° E, mean temperature 26 °C), Hainan province, China.

### Creation of transgenic overexpression plants of *GmFULa*

For the *GmFULa* overexpression construct, the full-length coding sequence of *GmFULa* from ZGDD was cloned into the binary vector pTF101.1 between the *XbaI* and *SacI* sites, downstream of the constitutive Cauliflower Mosaic Virus 35S promoter (Yue et al. 2017). The transformation of soybean followed the method of affecting Agrobacterium-mediated using the cotyledonary node explant (Paz et al. 2004). Transgenic plants were verified by PCR-based markers with primers listed in Supplementary Table S1. The soybean transgenic lines were advanced to the T5 generation.

### Soluble sugar determination

The middle leaflet of the second fully expanded trifoliolate leaves in the V2 stage and the seed of R6 stage were used. The relative content of soluble sugar was determined with the Anthrone-sulfuric acid colorimetry method using the soluble sugar extract of ZGDD as reference (Huang et al. 2020).

### Sucrose synthase activity measurement

The sucrose synthase activity was measured with a Sucrose Synthase Activity Assay Kit (boxbio, Beijing, China). The relative sucrose synthase activity was expressed as the ratio between the sucrose synthase activities of transgenic lines and ZGDD.

### Chlorophyll concentration measurement

The middle leaflet of the second fully expanded trifoliolate leaves in the R2 stage (full blooming) were collected. The chlorophyll was extracted and measured with the method described by Xu et al. (2013).

### Biomass measurement

The whole plants of 3, 7 and 15 DAG were used and cut into separate shoot and root parts from the cotyledon node. Then, the shoot and root samples were sterilized at 105 °C for 30 min, dried to constant weight, and weighed.

### Photosynthesis rate analysis

For each group, fifteen plants were randomly selected. The middle leaflet of the second fully expanded trifoliolate leaves in the V3 stage was used. The photosynthesis measurement was conducted with the Li-6400 portable photosynthesis measuring system (LI-COR, USA) (Singsaas et al. 2004; Xu et al. 2013; Busch 2018). The photo-synthetically active radiation was set up as 1,200 µmol photons m^−2^ s^−1^ (Xu et al. 2013).

### Leaf morphology and anatomy

The middle leaflet of the third trifoliolate leaf from top was sampled for anatomy analysis, which was performed by Servicebio, China (https://www.servicebio.cn/). The sections were stained with safranin O and fast green (Langdale et al. 1989), mounted with neutral balsam and scanned with a Pannoramic 250 Flash II Scanner (3DHISTECH Kft., Budapest, Hungary). The thicknesses of the leaf, spongy mesophyll, stratum corneum and palisade, the numbers of veins and palisade tissue cells, and the cell sizes of the upper epidermis and palisade cells were evaluated at five points.

### Gene expression analysis

The middle leaflet of the second expansion trifoliolate leaves of soybean V3 stage was sampled. RNA of transgenic plants and wild-type ZGDD was isolated using TRIZol (ET111), and reverse transcribed into cDNA with *Easyscript®* one-step gDNA removal and cDNA synthesis superMix (AT311) (Transgen Biotech, Beijing, China). The cDNA concentrations were normalized to the *GmActin* expression levels for quantitative PCR analysis (Quant Studio™ 12 K Flex). The qPCR primers are listed in Supplementary Table S1. The primer specificity and efficiency verification used primer blast in NCBI (https://www.ncbi.nlm.nih.gov/). PCR cycle conditions as hold stage: 95 °C 20 s, then PCR stage: (95 °C 1 s, 60 °C 20 s) X 50 cycle, and melt curve stage: 95 °C 15 s, 60 °C 1 min, 95 °C 15 s. The relative expression levels were estimated using the 2^−△△CT^ method (Taylor et al. 2019). Three biological replicates were included.

### Transient expression in soybean protoplasts

The *GmFULa* CDS without the stop codon was amplified and fused to the 5’ end of the open reading frame encoding GFP in pTF101 (Yue et al. 2017), which was driven by the *CaMV35S* promoter. For the ProGmSUS12YFP construct, a genomic DNA sequence (from − 1224 to − 1 bp) upstream of the *GmSUS12* coding sequence was amplified using sequence-specific primers and the sequence was cloned into *Kpn*I and *Xho*I sites of the pYFPLT vector, which contains the yellow fluorescent protein (YFP) coding sequences. The recombinant construct was transformed into soybean protoplasts.

Selected 14-day soybean leaves were cut into 1 mm strips and incubated with the enzyme digestion solution (1% Cellulase “Onozuka” Rs, 0.5% Pectolase Y-23, 9% Mannitol) in the dark for 5 h at 25 °C. An equal volume of CPW9M solution was added (Frearson et al. 1973). Digested tissues were filtered through a molecular sieve with 100 mesh number. After centrifugation at 200*g* for 5 min at 4 °C, the protoplast was washed and precipitated three times with CPW9M. Plasmid (10 µg) was added to 100 µL protoplasts, which were resuspended in MMG and incubated with 110 µL PEG4000 solution for 15 min at 25 °C. After that, 600 µL W5 stop solution was used to end the transfection the protoplasts were washed twice with CPW9M or W5 as described previously (Yoo et al. 2007). Finally, protoplasts were cultured in CPW9M for 20 h in the dark at 25 °C. Fluorescence images were taken using a confocal laser scanning microscope.

### Protein expression, purification and electrophoretic mobility shift assay (EMSA)

For expression of GmFULa protein in bacteria, the *GmFULa* full-length coding sequences were inserted into the inducible expression vector pET-32a (with 6 × His Tag) between *BamH*I and *Sac*I sites. The resulting plasmids were transformed into *Escherichia coli* strain Rosetta (DE3) and induced using 0.4 mM isopropyl-b-ß-1-thiogalactopyranoside (IPTG) at 25 °C for 12 h. The recombinant protein was purified using Proteinlso^R^ Ni–NTA Resin (Transgen biotech, Beijing, China) according to the manufacturer’s protocol. For *GmSUSs* and *GmSUTs*, the probe fragment consisted of a region of 40 bp with the canonical CArG box (C[A/T]_8_G) in the center (Table S1 for primer sequences). The mutated CArG box fragment TM was CCGCG (AATAT) in the mid region of the TG motif. The probes were labeled using the EMSA Probe Biotin Labeling Kit (Beyotime Biotechnology). The same fragments without biotin labeling were used as competitors. The protein–DNA complexes were separated with 6% native polyacrylamide gels. The Biotin-labeled probes were visualized using Chemiluminescent Biotin-labeled Nucleic Acid Detection Kit (Beyotime Biotechnology) according to the manufacturer’s protocol.

## Results

### *GmFULa* has two highly conserved, dominant haplotypes distributed in both wild and cultivated soybeans

**Fig. 1:**
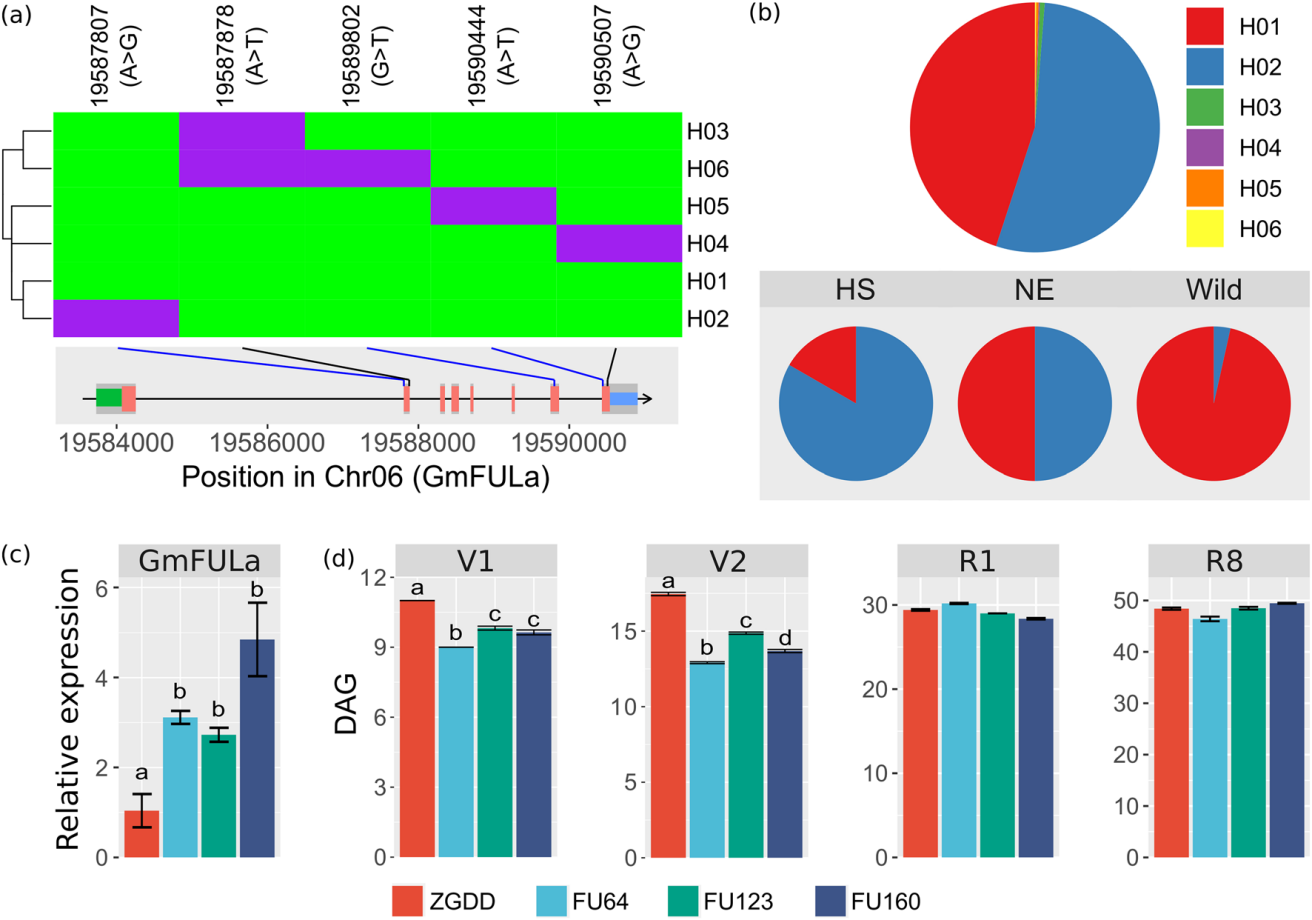
Polymorphisms and haplotypes of *GmFULa* and the effect of one dominant haplotype on maturity. **a** Polymorphisms and haplotypes of *GmFULa*. Bottom inset shows the exon–intron structure of *GmFULa* and the location of polymorphisms, where green, red and blue bars indicate 5’ UTR, CDS and 3’ UTR, respectively, and blue and black lines, respectively, indicate missense and synonymous variants. The upper panel shows the haplotypes of *GmFULa*. Reference alleles are in green; alternative alleles are in purple. **b** Distribution of *GmFULa* haplotypes. The upper panel shows the general distribution of *GmFULa* haplotypes detected in all soybeans. The bottom panel shows the distribution of H01 and H02 haplotypes in cultivars from Northeast China (NE), cultivars from the Huang-Huai-Hai valley region and South China (HS), and wild soybeans (Wild). **c** Verification of *GmFULa* overexpression in transgenic lines by real-time quantitative PCR analysis. *GmActin* was used as an internal control. Values are given as mean ± SE of three biological replicates with letters showing if there is a significant difference between groups (One-Way ANOVA; Tukey HSD test at, < 0.05). **d** The growth stages of transgenic soybean lines and control soybean ZGDD. V1, V2, R1 and R8 are soybean growth stages of one unrolled trifoliolate leaf, two unrolled trifoliolate leaves, beginning flowering and full maturity, respectively. ZGDD: control soybean Zigongdongdou (transgenic receptor). FU64, FU123 and FU160 are independent transgenic lines overexpressing *GmFULa*. The data represent the mean ± SE of ≥ 20 biological replicates with letters showing if there is a significant difference between groups (One-Way ANOVA; Tukey HSD test at, < 0.05)

Based on the public soybean resequencing data (NCBI: SRP020131, SRP062560, SRP045129 and PRJNA589345) from several whole-genome resequencing studies (Lam et al. 2010; Zhou et al. 2015; Zhang et al. 2019b), we analyzed the polymorphisms of *GmFULa* in 549 lines including 86 wild soybeans. We found 161 variation sites, of which there were 141 SNPs (single nucleotide polymorphisms) and 20 indels (insertions and deletions), including two synonymous mutation sites (black lines in Fig. 1a) and three missense mutation sites in the CDS (coding sequence) region (blue lines in Fig. 1a). Based on these five CDS variations, *GmFULa* was divided into six haplotypes: H01–H06, of which H01 and H02 were the most dominant (Fig. 1b). Moreover, in wild soybean, H01 was nearly the only haplotype. However, H01 and H02 were distributed nearly equally in the 80 widely planted cultivated soybeans from Northeast China, while H02 was dominant in the 54 widely planted cultivated soybeans from the Huang-Huai-Hai region and Southern China (Fig. 1b). These results indicated that H02 is an elite haplotype related to soybean geographical adaptation. Combined with the observation that *GmFULa* was highly expressed in the shoot apices (Jia et al. 2015), which strongly indicates that *GmFULa* should have an important role in yield. However, its actual function is unclear.

### *GmFULa* promotes soybean vegetative growth without affecting maturity

As GmFULa has three highly conserved homologs in soybean genome, and especially GmFULa is nearly identical to GmFULb with the identity rate as 92.2% in amino acid sequence and 94.1% in nucleotide sequence, respectively (Supplementary Figure S1), it is reasonable that *GmFULa* nullification through CRISPR/Cas9-based gene editing system will be compensated by these homologs especially *GmFULb*. Thus, to further evaluate the function of *GmFULa*, we only conducted a conventional overexpression analysis at this time. We cloned *GmFULa* from soybean variety Zigongdongdou (ZGDD, with the haplotype of *GmFULa-H02*) and constructed several transgenic overexpression lines where *GmFULa* was driven by the *CaMV35S* promoter. Homozygous transgenic lines were screened and identified by the combination of an herbicide test and PCR identification each generation (Supplementary Figure S2a and b). As expected, the expression levels of *GmFULa*, examined by quantitative PCR (qPCR), were significantly higher in the transgenic plants (FU64 and FU123, *p* < 0.01; FU160, *p* < 0.05) than in the control ZGDD (Fig. 1c). However, when determining whether GmFULa promotes plant growth, we found that although the transgenic and control plants reached the vegetative stages VE (emergence) and VC (unifoliolate leaves unrolled) on the same day, days to the subsequent vegetative stages V1 (one trifoliolate leaf unrolled) and V2 (two trifoliolate leaves unrolled) of transgenic lines were, respectively, about 2 days and 5 days shorter than those of the control (*p* < 0.01). In contrast, the days to the reproductive stages R1 (first flowering) and R8 (full maturity) of transgenic lines were nearly equal to those of the control (Fig. 1d). These results indicated that *GmFULa* neither affected germination nor maturity but promoted vegetative growth.

### GmFULa enhances the accumulation of biomass in a robust way

**Fig. 2:**
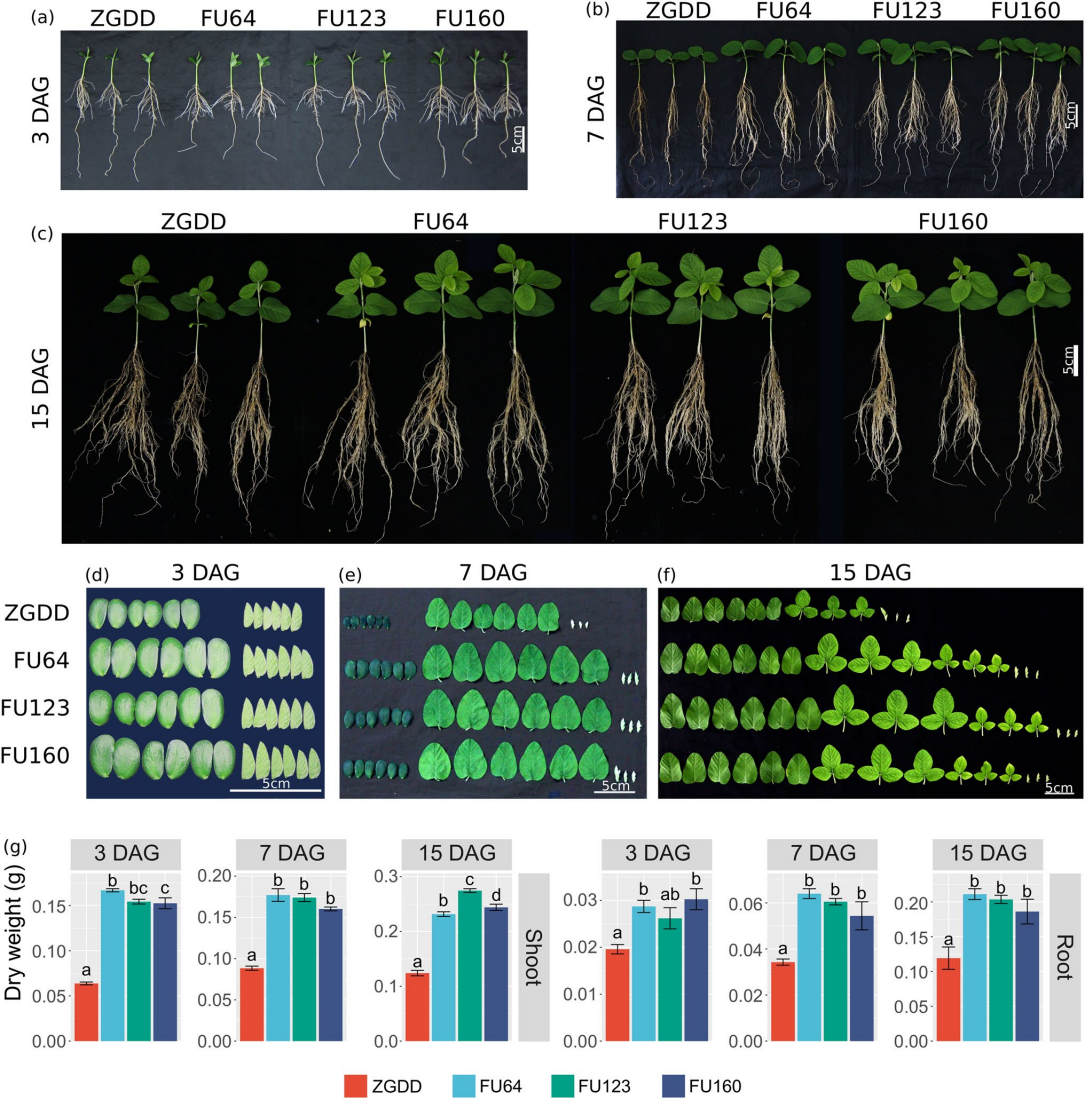
Overexpression of *GmFULa* enhances biomass accumulation. **a** Transgenic plants overexpressing *GmFULa* reach the same growth stage as control soybean ZGDD at 3 days after germination (DAG). **b** Transgenic plants overexpressing *GmFULa* have bigger unifoliolate leaves than control soybean ZGDD at 7 DAG. **c** Transgenic plants overexpressing *GmFULa* have one more trifoliolate leaf than control soybean ZGDD at 15 DAG. **d**–**f** Cotyledons and leaves of transgenic plants overexpressing *GmFULa* and control soybean ZGDD at 3 (**d**), 7 (**e**), and 15 (**f**) DAG. **g** Overexpression of *GmFULa* promotes the accumulation of dry biomass at 3, 7 and 15 DAG (*n* = 5). ZGDD: control soybean Zigongdongdou (transgenic receptor). FU64, FU123 and FU160 are independent transgenic lines overexpressing *GmFULa*. Bar indicates 5 cm. The data represent the mean ± SE of five biological replicates with letters showing if there is a significant difference between groups (One-Way ANOVA; Tukey HSD test at, < 0.05)

Consistent with our previous observation, in terms of whole plants, transgenic lines had the same number of nodes and leaves as the control ZGDD at 3 and 7 DAG (days after germination), while they had one more node and trifoliolate leaves at 15 DAG (Fig. 2a–c). Wild-type plants reached the vegetative stage of V1 at 15 DAG, while transgenic lines had already entered the next stage of V2 (Fig. 2c), indicating that transgenic lines are more vigorous than the wild-type soybean. Moreover, for the three observation time points (3, 7, and 15 DAG), vegetative organs (cotyledons and leaves) were significantly bigger in transgenic soybeans than the wild-type control (Fig. 2d–f). Consistently, transgenic lines accumulated significantly higher dry biomass of both shoot and root compared to wild-type plants at 3, 7 and 15 DAG (Fig. 2g). These results suggested that *GmFULa* promotes vegetative biomass accumulation in soybean by increasing the vigor of plants.

### GmFULa regulates soybean sink content with increasing soybean yield

**Fig. 3:**
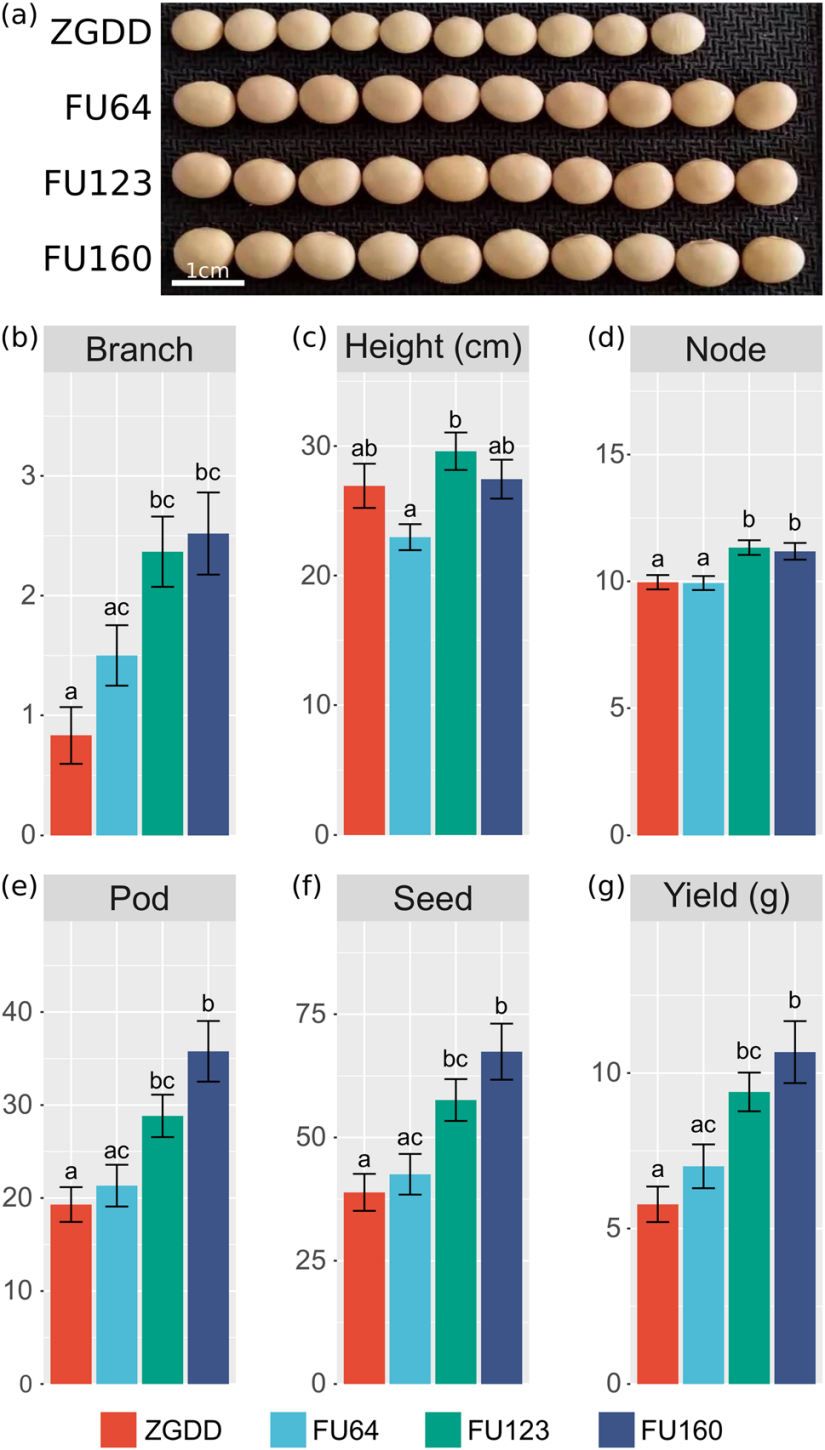
Overexpression of *GmFULa* promotes soybean yield. **a** Representative seed sizes of transgenic lines and control. **b**–**g** Yield-related traits branch number (**b**), plant height (**c**), node number (**d**), pod number (**e**), and seed number (**f**) and overall yield (**g**) of transgenic lines and control grown under natural field conditions. The plants were grown in a field in Sanya, Hainan province, China. The data represent the mean ± SE from three replicates (ten plants per replicate) with letters showing if there is a significant difference between groups (One-Way ANOVA; Tukey HSD test at, < 0.05). ZGDD: control soybean Zigongdongdou (transgenic receptor). FU64, FU123 and FU160 are independent transgenic lines overexpressing *GmFULa*

Although GmFULa promotes vegetative growth and biomass accumulation leading to increase the source capacity at the vegetative stage, it is necessary to further confirm whether GmFULa can promote the transformation of carbohydrate source–sink to increase soybean yield. To correspond with actual production practices, we evaluated the yield potentiality of *GmFULa* under natural field conditions. Transgenic overexpression lines (FU64, FU123, FU160) and wild-type control (the transgenic receptor cultivar ZGDD) were grown in an experimental field in Sanya, Hainan province. Yield-related traits were investigated, including the branch number, plant height, node number, pod number, and seed number as well as overall yield. In keeping with the results of the experiments performed in incubators, the *GmFULa*-overexpression plants grew better compared to the wild-type control with more pods and more branches but similar height (Fig. 3). The seed sizes of transgenic lines were also bigger than the wild type (Fig. 3a). Consistently, the yield-related traits of branch number, pod number, and seed number as well as overall yield all increased significantly (*p* < 0.01) in all transgenic lines and the node number also increased in two transgenic lines (Fig. 3b–g). However, plant height did not show a significant difference between transgenic lines and wild-type control (Fig. 3c). Moreover, FU160 had the highest *GmFULa* expression level among the three overexpression lines; similarly, it had the highest values of yield-related traits branch number, node number, pod number, and seed number and overall yield. These results indicated that *GmFULa* increased soybean source content and promoted the source–sink transformation to increase soybean yield. No significant difference was observed in plant height between the overexpression lines and the wild-type control (Fig. 3c), suggesting that *GmFULa* is a candidate gene for ideal plant architecture with shorter node spacing that is more conducive to the utilization, transportation and storage of energy and materials from the source organ.

### *GmFULa* modifies the cell distribution of source organ leaf

**Fig. 4:**
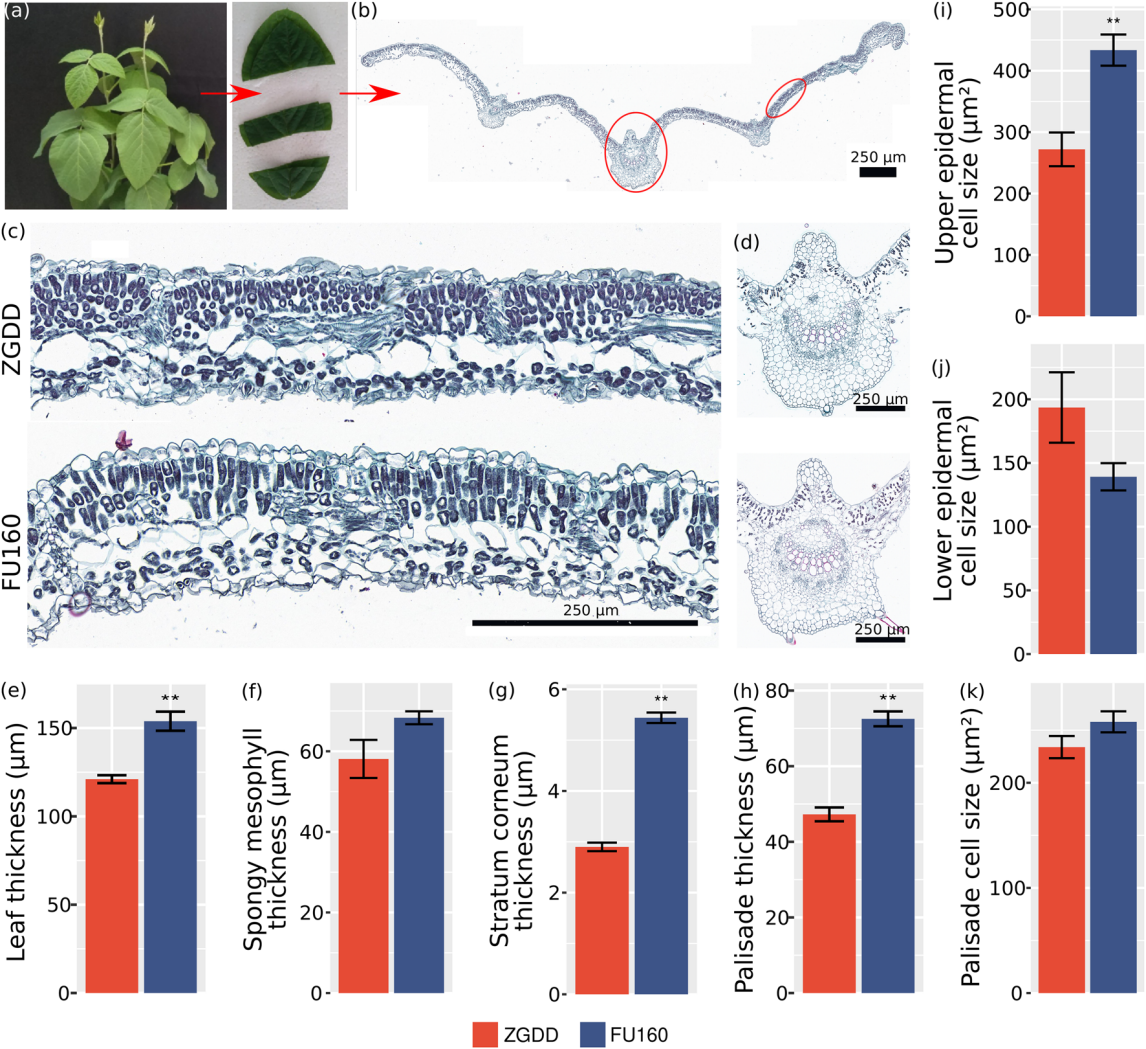
Overexpression of *GmFULa* increases the number and size of leaves cell. **a**, **b** Leaf sampling method for cell morphology analysis. **c** Cell morphology of mesophyll of cross section in wild-type and transgenic plants. **d** Cell morphology of leaf vein cross section in wild-type and transgenic plants. **e**–**k** Comparison of cell morphology in different tissues. ZGDD: control soybean Zigongdongdou (transgenic receptor). FU160 is the typical transgenic line overexpressing *GmFULa*. The data represent mean ± SE from three replicates. **p < 0.01 (Student's *t*-test). Scale bars are 250 μm

To understand how *GmFULa* regulates yield-related traits, we further investigated how leaves, a major source organ, were changed by GmFULa overexpression. Various experiments provided direct hints that *GmFULa* increases leaf size and thickness, so the potential role of *GmFULa* in the shape and morphology of leaf cells should be clarified. We performed microscopic observations of leaf transects of a middle leaflet at the same trifoliolate node 25 DAE (days after emergence) of the typical overexpression line FU160 and the wild-type control ZGDD (Fig. 4a, b). Consistently, the leaf of the transgenic line was significantly thicker than that of the wild-type control (Fig. 4c–e). The numbers of veins and palisade tissue cells in transgenic lines increased significantly compared to those of the wild-type control (Supplementary Table S2 and Fig. 4d). The spongy mesophyll, stratum corneum and palisade were thicker in the transgenic line than in the wild-type control (Fig. 4f–h), though the difference was not significant for spongy mesophyll. In addition, the cell size of the upper epidermis (FU160 = 434 µm^2^, ZG = 272 µm^2^) increased significantly in transgenic line FU160 while the cell size of the lower epidermis decreased (Fig. 4i, j and Supplementary Table S2). Palisade cell size in FU160 was not significantly different from the one in control ZGDD (Fig. 4k). These data suggest that *GmFULa* has an important role in regulating cell distribution and palisade development in leaf photosynthesis.

### GmFULa regulates source–sink balance with carbon assimilation and transfer

**Fig. 5:**
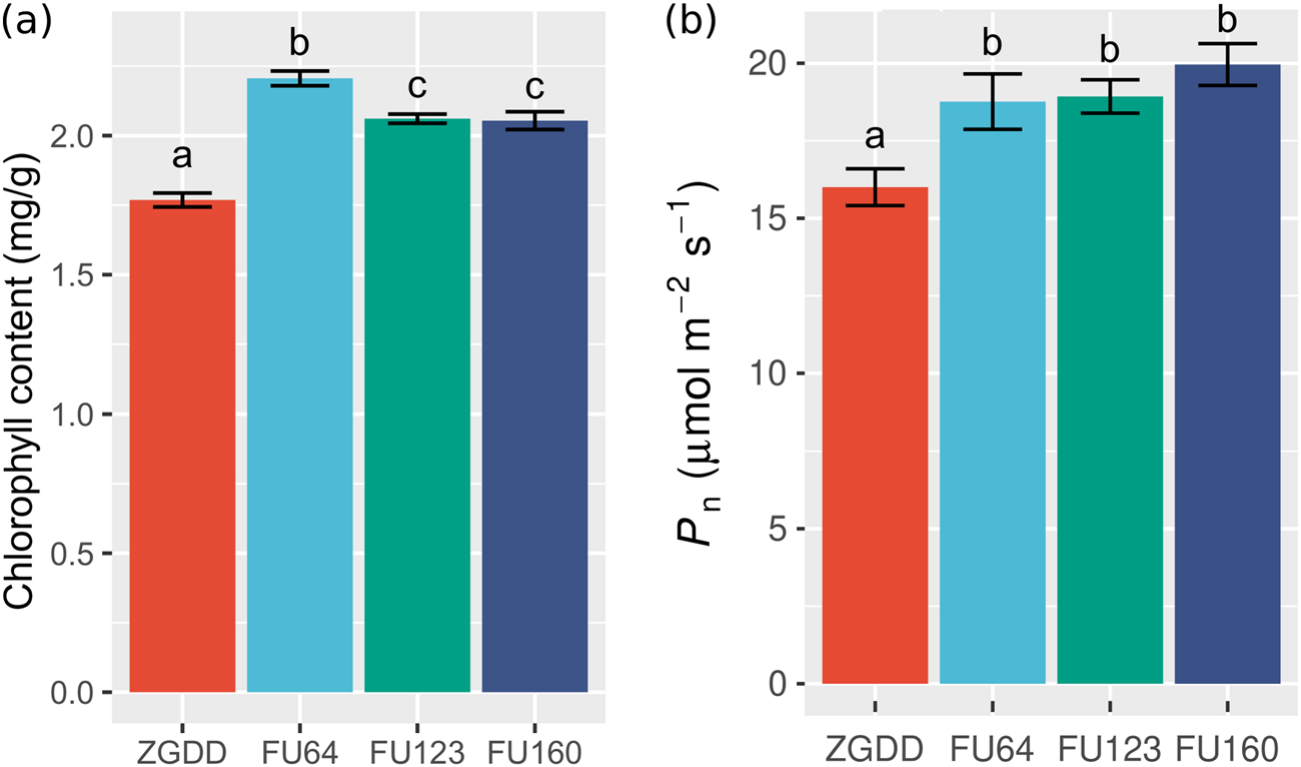
Overexpression of *GmFULa* increases the content of chlorophyll and promotes the rate of photosynthesis. **a** Chlorophyll content in control and transgenic plant leaves. The data represent mean ± SE from three biological replicates with letters showing if there is a significant difference between groups (One-Way ANOVA; Tukey HSD test at, < 0.05). **b** Photosynthesis in control and transgenic plants. ZGDD: control soybean Zigongdongdou transgenic receptor. FU64, FU123 and FU160 are the independent transgenic lines overexpressing *GmFULa*. The data represent mean ± SE from fifteen biological replicates with letters showing if there is a significant difference between groups (One-Way ANOVA; Tukey HSD test at, < 0.05)

**Fig. 6:**
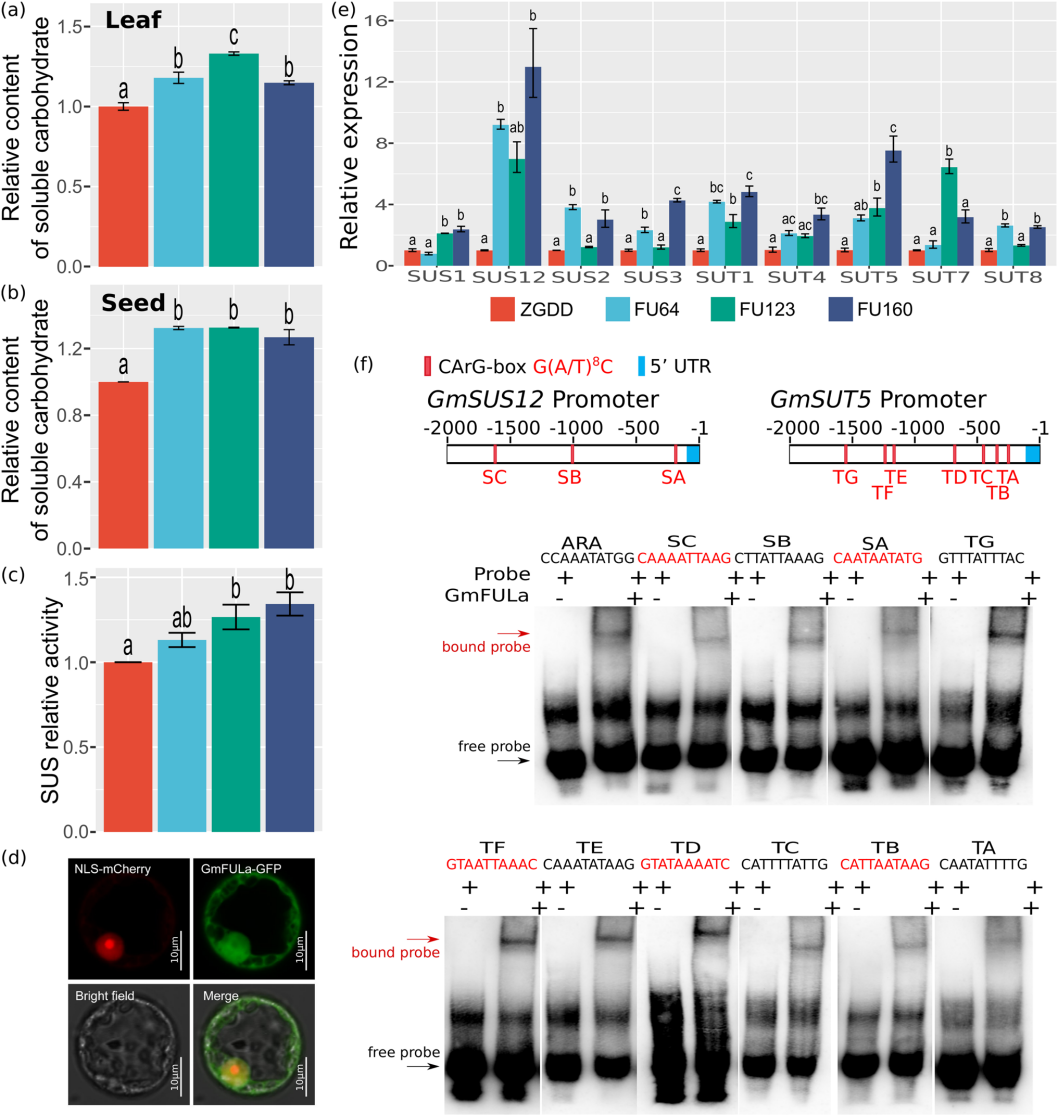
*GmFULa* regulates sucrose synthases and transporters to increase soluble sugar content of soybean leaves and seeds. **a** Relative content of soluble carbohydrate in leaves of transgenic lines and control plant. **b** Relative content of soluble carbohydrate in seeds of transgenic lines and control plant. **c** The activity of sucrose synthase in leaves of transgenic lines and control plant. **d** Subcellular localization of GmFULa in soybean protoplasts. GFP and GmFULa-GFP fusions under the control of the *CaMV35S* promoter were transiently expressed in soybean protoplasts. Bar = 10 µm. **e** Relative expression levels of *GmSUSs* and *GmSUTs* in transgenic lines and the control. Soybean *GmActin* was used as an internal control. **a–c**, **e** The data represent mean ± SE from three biological replicates with letters showing if there is a significant difference between groups (One-Way ANOVA; Tukey HSD test at, < 0.05). **f** *GmFULa* binds to the *GmSUS12* and *GmSUT5* promoters. Schematic diagram of the 2,000 bp *GmSUS12* and *GmSUT5* promoter regions showed three and seven CArG boxes, respectively. EMSA assay testing the binding of *GmFULa* to the *GmSUS12* and *GmSUT5* promoter fragments. Two 40 bp single-strand oligonucleotide probes containing CArG box motif with 16 bp flanking sequences were synthesized and labeled with biotin. + and – indicate the presence and absence of the corresponding probe or protein. ZGDD: control soybean Zigongdongdou (transgenic receptor). FU64, FU123 and FU160 are the independent transgenic lines overexpressing *GmFULa*

To study the physiological function of *GmFULa* in soybean growth, we determined chlorophyll content, photosynthesis rate, soluble sugar content and sucrose synthase activity. For chlorophyll content, leaf blades were sampled with a hole punch from the middle leaflet of the second fully expanded trifoliolate leaves of three plants in the V2 stage and weighed. The content of chlorophyll of overexpression plants were significantly higher than those of the wild-type control (Fig. 5a). Correspondingly, the leaf-level photosynthesis rates of transgenic plants were also significantly enhanced in the field conditions (Fig. 5b). The content of soluble sugar in leaves of V2 stage and seeds of R6 stage were further detected. Compared to WT, transgenic overexpression lines exhibited significantly higher levels of whole-soluble sugar both in leaves and seeds (Fig. 6a, b). Consistently, the activity of sucrose synthase of transgenic overexpression lines was higher than that of the wild-type control, though this difference was only significant for FU160 (Fig. 6c). These physiological data indicated that *GmFULa* enhances assimilation in soybean.

### GmFULa binds to the conserved CArG boxes present in the promoter regions of *GmSUS12* and *GmSUT5*

To understand how *GmFULa* regulates agronomic and physiologic traits, we further analyzed its protein subcellular localization. By transient expression of GmFULa-GFP (green fluorescent protein) driven by *CaMV35S* promoter in soybean protoplasts, we found that the fusion protein was localized in the nucleus of soybean protoplasts based on the observation that the GFP signal was exclusively co-localized with the mCherry-labeled nuclear signal (Fig. 6d). These results were in line with the prediction that GmFULa should be a transcription factor.

Considering that soluble sugar synthesis was promoted in the transgenic lines, it is a reasonable hypothesis that *GmSUSs* and *GmSUTs* should be regulated by GmFULa. To clarify this hypothesis, we first performed a qPCR experiment to compare the expression of 12 *GmSUSs* and eight *GmSUTs*, and found that most *GmSUSs* and *GmSUTs* (especially *GmSUS12* and *GmSUT5*) had significantly higher expression in overexpression lines FU64, FU123 and FU160 leaves of V3 stage than in control plant ZGDD. This result suggests that *GmSUS* and *GmSUT* are regulated by *GmFULa* in soybean growth (Fig. 6e).

To further confirm whether *GmFULa* regulates *GmSUS* and *GmSUT* directly, *GmSUS12* and *GmSUT5* were selected for further analysis. In the 2,000 bp upstream promoter region of both *GmSUS12* and *GmSUT5*, we found three (SA–SC) and seven (TA–TG) FUL-combining CArG boxes, respectively (Fig. 6f), which indicated that GmFULa might combine these boxes to regulate the expression of *GmSUS12* and *GmSUT5*. Then we performed an EMSA, and found a shift for all detected CArG boxes (SA–SC and TA–TG) as predicted, confirming that GmFULa can physically bind to the promoters of *GmSUS12* and *GmSUT5* (Fig. 6f). Furthermore, a yeast one hybrid experiment also showed that GmFULa binds to GmSUS12 promoter. These results indicate that GmFULa promotes the activity of sucrose synthesis and transport-related genes in soybean.

## Discussion

*FRUITFULL* (*FUL*), a MADS-box transcription factor, is essential in the network that regulates the initiation of shoots and buds, the transformation of reproductive growth and the development of organs. In *Arabidopsis thaliana*, *FUL* down-regulates *AP2* (*APETALA2*) and *IND* (*INDEHISCENT*) and promotes pod elongation (Di Marzo et al. 2020). In cucumber, *CsFUL1* regulates fruit length (Zhao et al. 2019). *DEFH28* from *Antirrhinum majus* regulates carpel wall differentiation and fruit ripening (Müller et al. 2001). Bemer et al. (2012) found that *TDR4*/*FUL1* and *MBP7*/*FUL2* affected fruit ripening independent of ethylene. Similarly, our previous study found that *GmFULa* is specifically expressed in flowers and pods, and is related to photo-thermal adaptation of soybean (Jia et al. 2015). However, we knew less about the exact role of *GmFULa* on soybean maturity and yield and how it works.

Using publicly available whole-genome resequencing data, we found that *GmFULa* had two major haplotypes, *GmFULa-H01* and *GmFULa-H02*. The proportion of *GmFULa-H02* is highest in cultivated soybeans from Middle and South China and second highest in cultivated soybeans from Northeast China, but nearly absent in wild soybeans. These results indicate that *GmFULa-H02* is an elite allele to be highly selected during domestication and improvement of cultivated soybeans and might promote the expansion of soybeans from high latitudes to low latitudes.

Different from our expectation that *GmFULa* might regulate soybean maturity and improve soybean adaptation, *GmFULa* overexpression does not alter the maturity structure, or the durations of vegetative and reproductive growth, which is similar with *FUL* (*AGL8*) of Arabidopsis and *AaFUL1* of *Anthurium* (Gu et al. 1998; Ma et al. 2019). However, the *FUL* homologs in *Medicago truncatula* and *Platanus acerifolia* promote flowering (Jaudal et al. 2015; Zhang et al. 2019a). In combination with the observation that *GmFULa* promotes vegetative growth, GmFUL might function in a novel mechanism, as its ancestral homolog Dt2 does in determining semi-determinacy (Liu et al. 2016).

*GmFULa* has an important role in plant architecture through regulating the branch number, node number, leaf size, and leaf thickness. However, *GmFULa* had no significant effect on plant height when overexpressed. Moreover, it promoted the dry mass accumulation of both root and shoot and ultimately increased yield. In contrast, *GmAP1a* was found to control flowering time and plant height (Chen et al. 2020). These results indicate that *GmFULa* has pleiotropic roles on soybean growth and development. *GmFULa* promotes soybean yield, which increases adaptation. It is significantly different from known maturity loci, which regulate soybean maturity to improve soybean adaptation.

*GmFULa* regulates the source–sink balance. Before reproduction, *GmFULa* promotes vegetative growth and increases biomass accumulation, as indicated by the observations that chlorophyll content, photosynthesis rate, sucrose synthase activity, soluble sugar content, node number and branch number were all increased in transgenic soybeans compared to the wild-type soybean. However, considering that the number and size of seeds and the final yield were increased in transgenic soybeans compared to the wild-type soybean, *GmFULa* effectively promotes the source–sink transition during reproduction. Although the photosynthesis rates were smaller than the ones in Koester et al. (2016), our results were consistent with Lin et al. (2015) for the wild-type soybean Zigongdongdou. It might be possibly due to the genetic background difference: different from the soybeans in Koester et al. (2016) which are all maturity group III cultivars, Zigongdongdou is a MGX cultivar. Moreover, both the chlorophyll content and the photosynthesis rate were improved, which is consistent with the observation that photosynthesis rate is highly correlated with chlorophyll contents (Buttery and Buzzell 1977). Considering that the enhancement of leaf-level photosynthesis benefited from *chl* mutant did not necessarily resulted in canopy-level improvement (Slattery et al. 2016, 2017), it is necessary to further explore the effects of GmFULa on canopy-level processes.

**Fig. 7:**
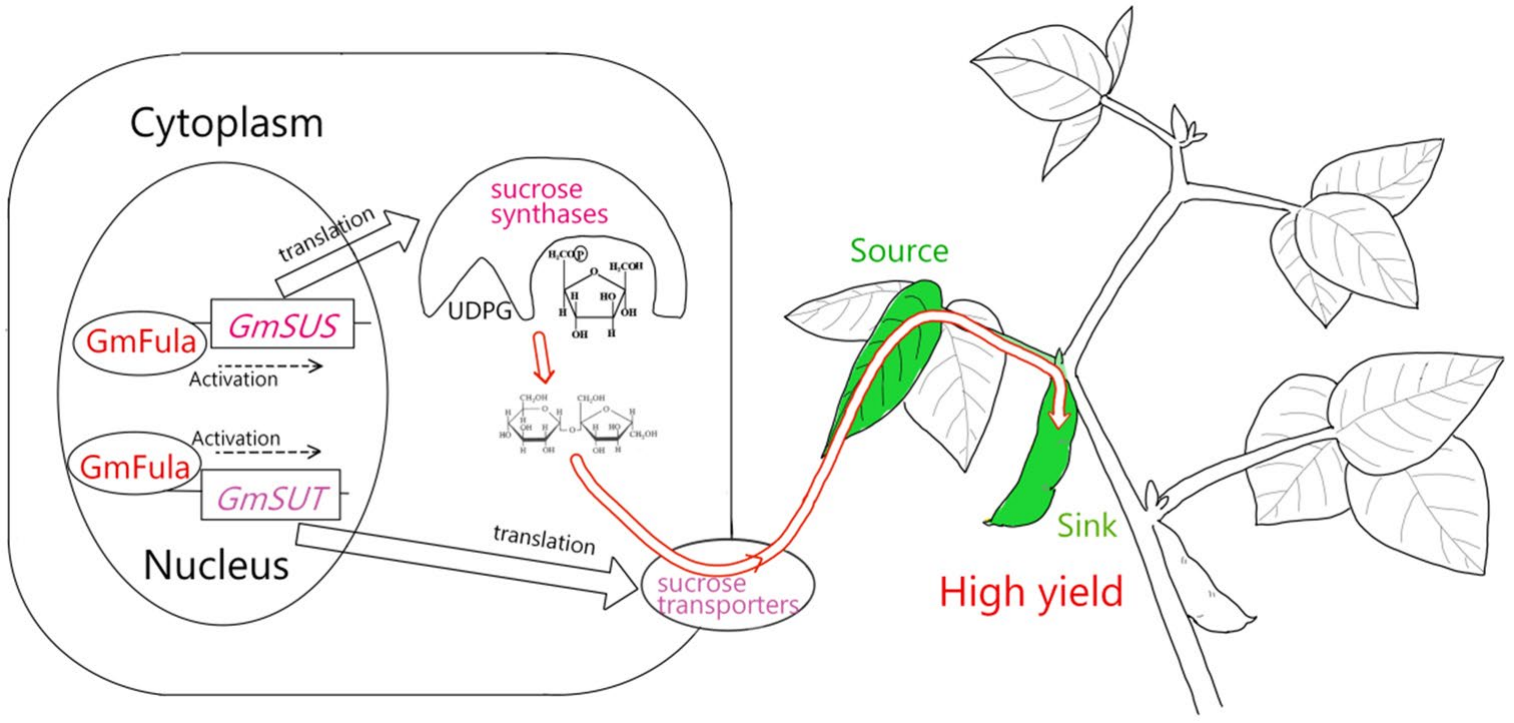
Working model for regulation of soybean vegetative growth, cell development, and yield by *GmFULa*. GmFULa regulates both the sucrose synthases (SUS) and the sucrose transporters to synchronize the sucrose biosynthesis (energy generation and assimilation) in the source organ (leaf) and the sucrose transportation (energy transportation) to the sink organ (pod) to finally promote yield

More importantly, consistent with our observation that *GmFULa* regulates the source–sink balance, it also regulates the expression of the sucrose synthase *GmSUS12* and the sucrose transporter *GmSUT5* through binding to their promoters. SUS is a key enzyme of sucrose metabolism, with an important role in the process of yield formation (Gessler 2021). Overexpressing potato *SUS* gene can increase cotton yield significantly (Xu et al. 2012). Moreover, it is also reported to be related to caryopsis development in rice, nitrogen fixation in legumes and plant response to stresses (Huang et al. 1996; Arrese-Igor et al. 1999; Xiao et al. 2014). SUTs are a kind of typical membrane binding protein, which are widely distributed in various tissues and organs of higher plants (Barker et al. 2000; Williams et al. 2000). They are responsible for the transmembrane transport of sucrose, and have a significant role in sucrose entering and leaving phloem, sucrose storage and sucrose supply to the sink tissues (Breia et al. 2020; Wang et al. 2020). Combining the increase in soluble sugar in leaves and seeds in transgenic lines, a working model of *GmFULa* was proposed (Fig. 7). In the model, GmFULa regulates the expression of SUS to promote sucrose biosynthesis in the source organ leaf. GmFULa also regulates the expression of SUT to promote sucrose transportation into the sink organ pod. Thus, GmFULa can synchronize both sucrose biosynthesis and transportation to increase the source–sink transition rate of the photosynthesis product and the photosynthesis rate is promoted as a result, which is consistent with the hypothesis proposed by Ainsworth et al. (2004). Moreover, as indicated by the observation that FUL homologs promote pod development, GmFULa efficiently promotes the utilization of sucrose in the sink organ pod to increase the final yield suggesting that GmFULa might also function in pod development. Moreover, GmFULa promotes root development, which means that more nutrients can be absorbed to support development of the above ground parts of plants.

*GmFULa* provides a new option for yield improvement of soybean. Soybean is a short-day crop that is sensitive to photoperiod, and the yield is highly dependent on adaption to local photo-thermal environments (Yue et al. 2017). Thus, in the breeding history of soybean, the first trait to address is photoperiod sensitivity, specifically, modifying maturity to match the soybean photoperiod sensitivity to local environments. Multiple soybean maturity loci have been identified. With the discovery and application of maturity loci, soybean has expanded to higher and lower latitudes, resulting in huge increases in soybean production. If the production increase mainly resulting from maturity adaption can be called the first-generation revolution of soybean breeding, the second-generation revolution of soybean breeding will be the improvement of traits other than maturity. In current breeding programs/strategies, the parental lines with different genetic backgrounds of maturity loci are challenging in conventional hybrid breeding, requiring efforts to find optimal photo-thermal environments for their filial lines through field experiments in different locations. Many known yield loci are highly linked to maturity traits; thus, transgenic modification of their causal genes might also have secondary effects that alter the maturity trait, and as a consequence, the main effect of yield will be uncertain if the optimal photo-thermal environments are changed. However, because *GmFULa* can improve the yield without altering the maturity, it is possible to directly improve elite cultivars without changing maturity. For an elite cultivar, we can modify the expression of *GmFULa* through transgenic overexpression or through gene editing to introduce an enhancer element, remove an inhibitor element or make a haplotype shift, consequently enhancing the yield capacity without significantly changing the optimal ecoregion.

In summary, we have functionally characterized *GmFULa*, a member of the MADS-box family in soybean. *GmFULa* has pleiotropic roles in soybean growth and development. It increases soybean adaptation through promoting vegetative growth and reproductive growth to increase soybean yield. It promotes source accumulation and the sink transformation, but does not affect maturity. Overexpression of *GmFULa,* thus, provides a new way to increase soybean yield and soybean adaptation.

## Supplementary Information

Below is the link to the electronic supplementary material.

Supplementary file1 (DOCX 815 KB)

## Abbreviations

CDS: Coding sequence
*DAG*: *Day after germination*
FUL: FRUITFULL
*SUS*: *Sucrose synthase*
*SUT*: *Sucrose transporter*
*ZGDD*: *Zigongdongdou*
