# Supplementary material for: GmFULa improves soybean yield by enhancing carbon assimilation without altering flowering time or maturity
Source: Plant Cell Rep. 2021 Jul 16;40(10):1875–88. doi: 10.1007/s00299-021-02752-y (PMC8494661; doi:10.1007/s00299-021-02752-y)
Supplement: Supplementary file 1 — Supplementary file1 (DOCX 815 KB) [file 299_2021_2752_MOESM1_ESM.docx]

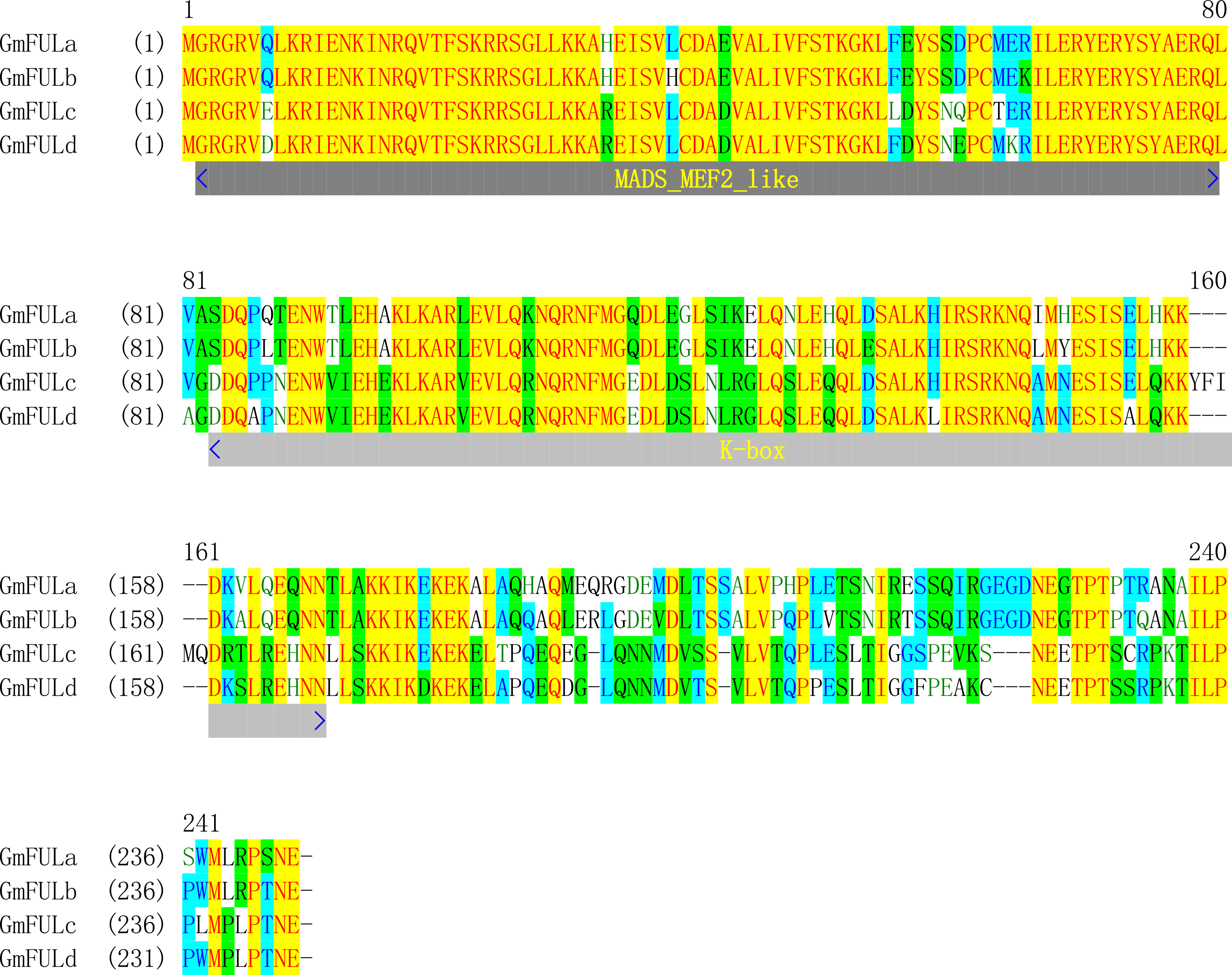


**Supplementary Figure 1. GmFULa are highly conserved with three homologs especially GmFULb.** MADS_MEF2_like and K-box conserved domains are shown in a dark-grey bar and a grey bar, respectively. Identical, conserve, and similar amino acids are colored by yellow, light-blue and green.


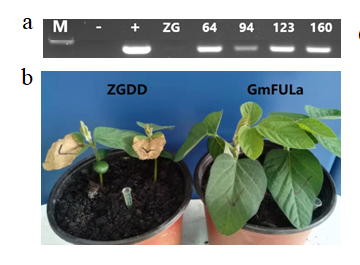


**Supplementary Figure 2. Identificationof transgenic plants.** (a) PCR detection of independent transgenic events. ZG, wildtype Zigongdongdou (transgenic receptor). 64, 94,123 and 160 are transgenic events of FU64, FU94, FU123 and FU160, respectively. M, DNA molecular standard. –, negative control (ddH_2_O). +, positive control (plasmid pTF101-GmFULa).(b) Screening of transgenic events with glufosinate. ZGDD, wildtype Zigongdongdou. GmFULa, a typical transgenic plant. All transgenic plants are overexpressing *GmFULa* under the control of the *CaMV35S* promoter.

**Supplementary table 1. Primers used in this study**

| **Primer name** | **Sequence (5’-3’)** |
| --- | --- |
| GmFULa-5 | TTGGATCCATGGGGAGAGGGAGAGTTCAGTTG |
| GmFULa-3 | TTGTAATCTTCATTTGAAGGACGAAGCATCCAAG |
| BamHI-FULA-F | CGCGGATCCatggggagaggaagggtgcagttga |
| FULAnotaa-SacI-R | CGAGCTCttcatttgaaggacgaagcatccaag |
| BAR506F | CTGAAGTCCAGCTGCCAGAA |
| BAR506R | ATGAGCCCAGAACGACGC |
| CaMV35S-F | GCTCCTACAAATGCCATCATTGC |
| CaMV35S-R | GATAGTGGGATTGTGCGTCATCCC |
| XbaI-Fula-F | GCTCTAGAATGGGGAGAGGAAGGGTGCAGTTGA |
| Fula3-GFP5-R | aacagctcctcgcccttgctcaccatTTCATTTGAAGGACGAAGCATCCAA |
| Fula3-GFP5-F | TTGGATGCTTCGTCCTTCAAATGAAatggtgagcaagggcgaggagctgtt |
| GFP3-FULA5-R | TTCAACTGCACCCTTCCTCTCCCCATCTTGTACAGCTCGTCCATGCCGAGA |
| GFP3-FULA5-F | tctcggcatggacgagctgtacaagATGGGGAGAGGAAGGGTGCAGTTGAA |
| FULA-SacI-R | cgagctcctattcatttgaaggacgaagcatcca |
| qACT11F | CGGTGGTTCTATCTTGGCATC |
| qACT11R | GTCTTTCGCTTCAATAACCCTA |
| SUS12AANTI | TTCATTATTTTCCTCCATATTATTGTCGTTTAATGAATAG |
| SUS12BSENSE | GTTAAGAAAACTCTTCTTATTAAAGATAACTAACTAAATT |
| SUS12BANTI | AATTTAGTTAGTTATCTTTAATAAGAAGAGTTTTCTTAAC |
| SUS12CSENSE | CTTATTAAGTAGCCACAAAATTAAGAGATTACTTTTAAAC |
| SUS12CANTI | GTTTAAAAGTAATCTCTTAATTTTGTGGCTACTTAATAAG |
| SUT5ASENSE | ACATTCAATGTCCAACAATATTTTGGTTAAAGTTACGTAG |
| SUT5AANTI | CTACGTAACTTTAACCAAAATATTGTTGGACATTGAATGT |
| SUT5BSENSE | AATCAATAAAGATTACATTAATAAGAGAATTAATTGGCCC |
| SUT5BNTI | GGGCCAATTAATTCTCTTATTAATGTAATCTTTATTGATT |
| SUT5CSENSE | AGAAACGGATACGTACATTTTATTGCATGTGACAAATATT |
| SUT5CANTI | AATATTTGTCACATGCAATAAAATGTACGTATCCGTTTCT |
| SUT5DSENSE | AATTAGGATAATTTTGTATAAAATCTATGATTTTAATTGT |
| SUT5DANTI | ACAATTAAAATCATAGATTTTATACAAAATTATCCTAATT |
| SUT5ESENSE | TGCTGTTTTTGGTGGCAAATATAAGAGACAGTTACCACAT |
| SUT5EANTI | ATGTGGTAACTGTCTCTTATATTTGCCACCAAAAACAGCA |
| SUT5FSENSE | ATAAAAATTAAATAAGTAATTAAACTTATTTTTTATATGA |
| SUT5FANTI | TCATATAAAAAATAAGTTTAATTACTTATTTAATTTTTAT |
| SUT5GSENSE | TTAATTATTTTTTAGGTTTATTTACTCATTTAAATACTAT |
| SUT5GANTI | ATAGTATTTAAATGAGTAAATAAACCTAAAAAATAATTAA |
| CARG-P-SENSE | ATGTTTCAATAATGTCCAAATATGGTAACAAAATACACGT |
| CARG-P-ANTI | ACGTGTATTTTGTTACCATATTTGGACATTATTGAAACAT |
| GmSUT1-F | CTGCTGCATTGACTTTCTTCTC |
| GmSUT1-R | CTCCTGAAGTGGTGGAGTATATG |
| GmSUT2-F | CAGGAGCAGGACAAGGTTTAT |
| GmSUT2-R | GGAATCCCAAGGACCACTTAAT |
| GmSUT3-F | GCAATAGTGGTCCCACAGATAA |
| GmSUT3-R | GTCCACTGATAAGGGCTGAAA |
| GmSUT4-F | GGACCATGGGATGCTCTATTT |
| GmSUT4-R | GGAGATGGCAGCAGGATTAT |
| GmSUT5-F | GCTATAAGTGGACAGTGGGATAAG |
| GmSUT5-R | AGCAGAACAACCGCTAACA |
| GmSUT6-F | CGCTTGGGCCTCATTTATTTG |
| GmSUT6-R | CAAAGACCCGGCTAGGATAAAG |
| GmSUT7-F | GTGGAAACTTGCCTGCATTC |
| GmSUT7-R | CCTGACCTCATCAGCTTTCTT |
| GmSUT8-F | GTCCTACAAAGGTCTCCACAAG |
| GmSUT8-R | AGCAGCAAGAGGATTGAGAAA |
| GmSUT9-F | TTCTTCAGGTGCTAGGGAAAG |
| GmSUT9-R | ACAGCAAGCTCTAGTGTCAAG |
| GmSUS1-F | ATATGCCTGATGCATTGCGG |
| GmSUS1-R | GAAGCCAAGAATGCCCTCCA |
| GmSUS2-F | GGCTCAGGGGAAAGGGATTT |
| GmSUS2-R | TGGACGAACTGCTATTGCCA |
| GmSUS3-F | TCACCAAGTGGTTGCAGAGTT |
| GmSUS3-R | GCCTTGGTCGAACAGCAAGA |
| GmSUS4-F | CCTTGAAGCAAAGCCGGTTC |
| GmSUS4-R | GGCCTCACTGCAAAAGCAAT |
| GmSUS5-F | TGGTACCATGTGAAGAGGTGC |
| GmSUS5-R | CAAGGTTCCCCTCCAACACT |
| GmSUS7-F | TGAGGCAGAGCCGGTATCAT |
| GmSUS7-R | AACAGCTTCCTGTGTGGAGC |
| GmSUS8-F | GCTGGGGGCAAAGGAATACT |
| GmSUS8-R | AGGTCTTGGACGCAAAGCTA |
| GmSUS9-F | AGGGAATCCTGCAACACCAC |
| GmSUS9-R | CCCGCAGATACTCCCAAACA |
| GmSUS11-F | CCACCAGGTCATTGCTGAGT |
| GmSUS11-R | CCTCAACAACAAGAGCGTGC |
| GmSUS12-F | AGAGGGATTTTGCAACCCCA |
| GmSUS12-R | AACACCAGGTCTTGGACGAA |
| GmSUS10-F | CAGATAGCATGCCAGAAGCCT |
| GmSUS10-R | CATATGGTGGAACAACAGCGG |

**Supplementary table 2. Cytological analysis**

| **Metrics** | | **ZGDD** | **OEGmFULa** |
| --- | --- | --- | --- |
| **Cell number** | **Upper epidermis** | 10±1.41 | 13±1.15 |
|  | **Lower epidermis** | 13±2.94 | 11.75±1.25 |
|  | **Palisade tissue** | 25.5±0.70 | 32±2.48 |
|  | **Spongy tissue** | 10.5±0.70 | 7.5±2.12 |
| **Cell area (µm²)** | **Upper epidermis** | 2963±498 | 2512±815 |
|  | **Lower epidermis** | 3351±221 | 2220±644 |
|  | **Palisade tissue** | 14942±4373 | 18050±3405 |
|  | **Spongy tissue** | 11150±3545 | 15726±6129 |
| **Thickness of the midrib (mm)** | | 0.89±0.07 | 0.84±0.09 |
| **Number of the veins** | | 2.5±0.70 | 2.5±0.70 |
| **Length of veins (mm)** | | 7.25±3.33 | 9.01±4.65 |
| **Number of veins per unit length(n/mm)** | | 0.41±0.29 | 0.34±0.26 |
